# From Gravity to Affinity: Transitioning From Penrose Drains to Dialkylcarbamoyl Chloride (DACC)-Coated Technology in the Adjunctive Management of a Deep Neck Infection

**DOI:** 10.7759/cureus.109114

**Published:** 2026-05-18

**Authors:** Deddy D Septian, Indra Hadikrishna, Melita Sylvyana

**Affiliations:** 1 Oral and Maxillofacial Surgery, Universitas Padjadjaran, Bandung, IDN

**Keywords:** antimicrobial stewardship, deep neck space infection, dialkylcarbamoyl chloride, penrose drain, phlegmon, wound infection

## Abstract

Deep neck space infections (DNSIs) are life-threatening emergencies requiring rapid surgical source control and systemic antibiotic therapy. While surgical decompression traditionally relies on passive Penrose drains, these drains do not actively manage local microbial bioburden. We report the case of a 72-year-old male presenting with an extensive odontogenic cervical phlegmon extending to the chest wall. Emergency incision and drainage was performed, and a Penrose drain was placed. Microbiological cultures of the purulent exudate grew *Staphylococcus aureus*. On post-operative day (POD) 1, given the high purulent output (15 cc), the drainage strategy was transitioned to a dialkylcarbamoyl chloride (DACC)-coated dressing to actively sequester local pathogens through hydrophobic interaction. Following application, purulent output dropped to 7 cc on POD 2 (a 53% reduction), and the wound completely resolved by POD 7. While the patient's primary recovery was undoubtedly driven by surgical decompression and systemic antibiotics, this single-case observation suggests that transitioning to a DACC-coated dressing represents a feasible and possible adjunctive option for managing high-volume exudate in the sub-acute phase of fascial infections.

## Introduction

Deep neck space infections (DNSIs) are notoriously aggressive. When these suppurative processes spread through the cervical fascial planes, the clinical picture can deteriorate rapidly, especially in geriatric patients who are already at a heightened risk for airway obstruction, descending necrotizing mediastinitis, and septic shock [1]. Because of this high morbidity, the standard of care requires a dual-pillar approach: securing the airway and performing immediate surgical decompression via incisional and drainage procedures for mechanical source control, coupled with the rapid initiation of broad-spectrum systemic antibiotics [2].

For decades, surgeons have relied on passive systems, such as the Penrose drain introduced in the late 19th century, for post-operative decompression [3]. There is no denying their efficiency in venting fluid and relieving immediate tissue pressure. Yet they are purely mechanical; they do not actively clear the residual bacterial load in the wound bed. This is where dialkylcarbamoyl chloride (DACC)-coated dressings present an interesting adjunctive option. Instead of actively lysing bacterial cells, which risks flooding the local tissue with proinflammatory endotoxins, DACC technology relies on hydrophobic interactions to physically bind and extract pathogens, safely managing high-volume microbial bioburden [4,5]. Here, we describe the management of an extensive *Staphylococcus aureus* cervical phlegmon, highlighting a clinical transition from initial passive drainage to the targeted use of DACC-coated technology.

## Case presentation

A 72-year-old male with no contributory systemic medical history presented with rapidly progressive, diffuse swelling of the right submandibular region, extending across the midline to the left mandible, the submental space, and inferiorly toward the anterior chest wall. The patient reported severe localized pain and odynophagia. Clinical examination revealed significant trismus, neck stiffness, hoarseness, and a "hot potato voice," signaling impending airway compromise. Immediate pre-operative laboratory evaluation revealed a severe systemic inflammatory response and impending tissue hypoperfusion, characterized by marked leukocytosis (WBC: 19.21 x 10^3^/uL), severe lactic acidosis (lactate: 17.0 mmol/L), and profound metabolic acidosis (pH: 7.380, pCO₂: 15.3 mm Hg, HCO₃: 9.1 mmol/L, base excess: -14.0 mmol/L). The diagnosis was an extensive cervical phlegmon secondary to the radicular involvement of teeth 45, 46, and 47.

**Figure 1:**
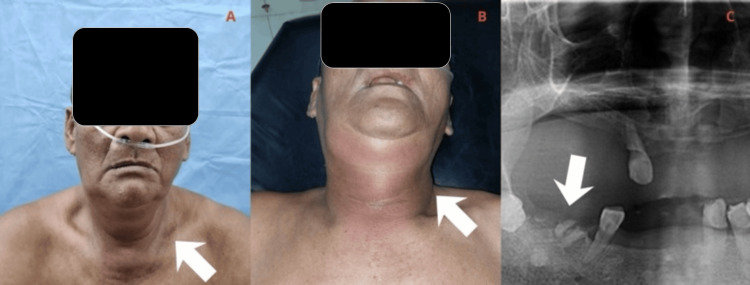
Pre-operative clinical and radiographic presentation A: anterior clinical photograph; B: submental view of the 72-year-old male demonstrating rapidly progressive, diffuse swelling and erythema originating from the right submandibular space and extending across the midline toward the anterior chest wall (arrow); C: cropped panoramic radiograph oriented to the right mandible, revealing severe dental caries and radicular involvement of the right posterior teeth (arrow), identifying the odontogenic source of the phlegmon. Written informed consent to include this image in an open-access article was obtained from the patient.

Emergency surgical decompression via wide incisional and drainage procedures was performed under general anesthesia. Multiple fascial spaces were explored, and a significant volume of malodorous, purulent material was evacuated. A sample of the purulent discharge was obtained during the procedure and sent for microbiological culture. A traditional passive Penrose silicone drain was placed to facilitate immediate mechanical decompression (Figure 1).

Empirical systemic antibiotic therapy was initiated with intravenous ceftriaxone (1 g twice daily) and metronidazole (500 mg three times daily), alongside standard intravenous analgesics to manage post-operative pain. The microbiological culture subsequently grew *Staphylococcus aureus*. Antibiotic susceptibility testing revealed the organism was resistant to penicillin G but sensitive to oxacillin, clindamycin, erythromycin, ciprofloxacin, and vancomycin, confirming a methicillin-sensitive *Staphylococcus aureus* (MSSA) infection.

**Figure 2:**
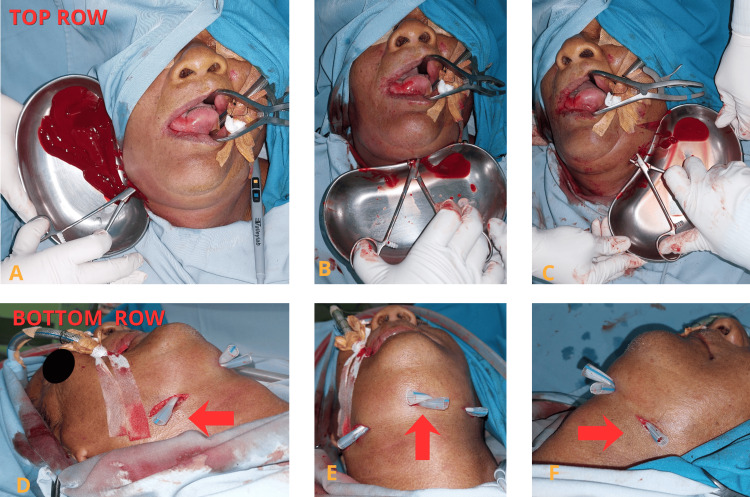
Incisional and drainage exploration A: surgical exploration oriented to the right submandibular space; B: the anterior submental space; C: the left submandibular space; D, E, F: subsequent placement of passive Penrose silicone drains (arrows) oriented within the right submandibular space, the submental space, and the left submandibular space, respectively, ensuring mechanical decompression of all involved deep fascial compartments. Written informed consent to include this image in an open-access article was obtained from the patient.

On post-operative day (POD) 1, the Penrose drain remained highly active, producing 15 cc of purulent discharge (Figure 2).

**Figure 3:**
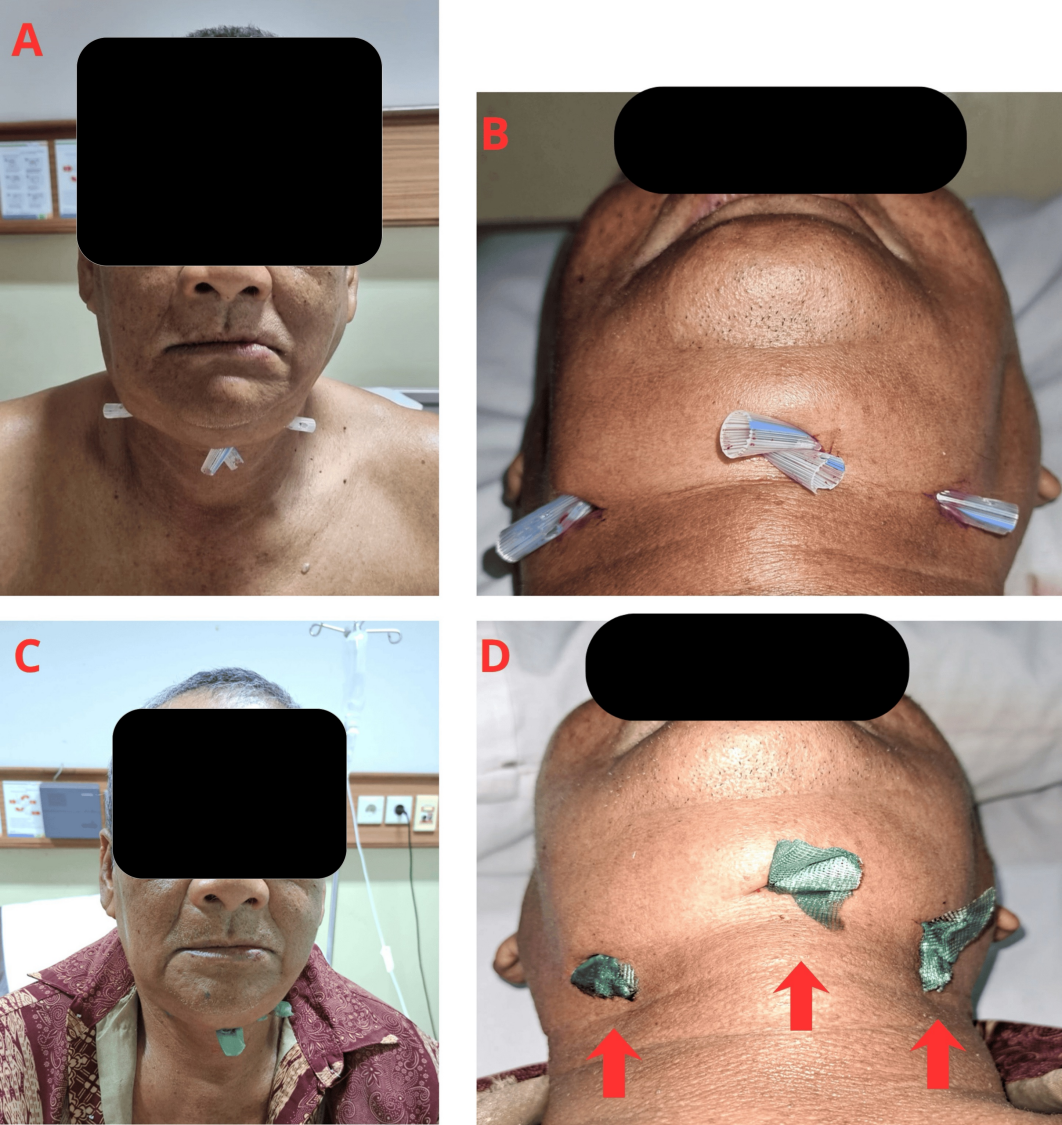
Transition of the drainage strategy on post-operative day 1 A: anterior view, B: submental view showing the highly active passive Penrose drains prior to removal; C: anterior view, D: submental view demonstrating the subsequent incremental packing of the dialkylcarbamoyl chloride (DACC)-coated ribbon dressings (arrows) oriented into the respective right submandibular, submental, and left submandibular fascial spaces to serve as active microbial-binding conduits following sterile wound irrigation. Written informed consent to include this image in an open-access article was obtained from the patient.

Given the continued high exudate volume and the goal of managing the local bioburden, the drainage strategy was transitioned. The Penrose drain was removed, and a sterile DACC-coated ribbon dressing, Cutimed Sorbact (Essity, Stockholm, Sweden), was incrementally packed into the fascial space using a sterile aseptic technique to serve as an active microbial-binding conduit (Figure 3). Purulent exudate volumes were subsequently measured daily using standardized collection vessels at fixed morning intervals to minimize reporting bias.

**Figure 4:**
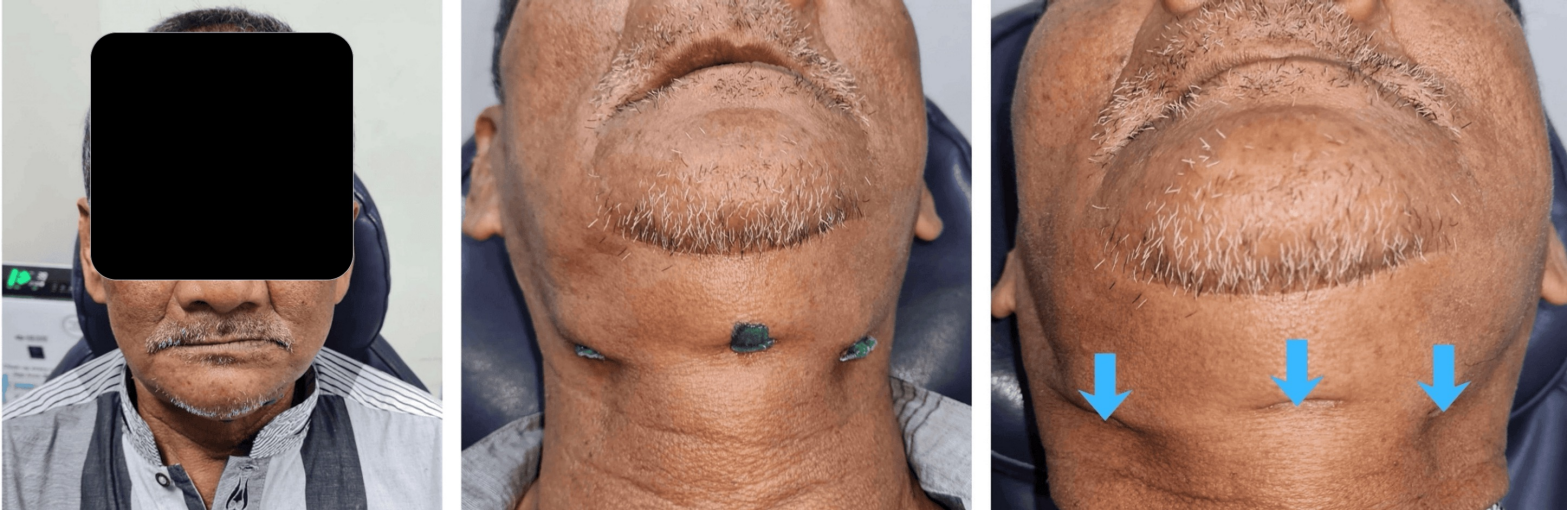
POD 5 and 7 clinical images A: anterior view; B: submental view on post-operative day (POD) 5 demonstrating marked resolution of the cervical phlegmon, with the dialkylcarbamoyl chloride (DACC)-coated dressings visible in the incision sites immediately prior to their final removal; C: submental view on POD 7 showing complete clinical resolution, absence of exudate, and healthy secondary intention healing with advanced granulation tissue at the drainage sites (arrows). Written informed consent to include this image in an open-access article was obtained from the patient.

By POD 2, purulent output dropped to 7 cc (a 53% reduction). Concomitantly, pharyngeal edema subsided, and the patient was deemed stable for discharge with oral cefadroxil and metronidazole, selected to provide continued broad-spectrum outpatient coverage against the isolated MSSA and potential oral anaerobes. On POD 5 in the outpatient clinic, production had reduced to 5 cc, and the DACC dressing was removed entirely (Figure 4A, 4B). At the POD 7 follow-up, the site showed complete clinical resolution (0 cc) with healthy secondary intention healing and advanced granulation tissue (Figure 4C).

The daily purulent drainage volume and corresponding clinical interventions are summarized in Table 1.

**Table 1:** Daily purulent drainage volume and clinical interventions DACC: dialkylcarbamoyl chloride

Post-operative Day (POD)	Drainage Volume (cc)	Clinical Intervention / Status
POD 1	15	Penrose drain removed; DACC-coated dressing applied
POD 2	7	Patient discharged; transitioned to oral antibiotics
POD 5	5	DACC-coated dressing removed
POD 7	0	Complete clinical resolution; healthy secondary healing

## Discussion

The management of extensive DNSIs fundamentally relies on the efficient removal of purulent material and the provision of suitable systemic antibiotics. In this instance, the swift stabilization of the patient was chiefly facilitated by the vigorous surgical decompression and the simultaneous systemic antibiotic treatment addressing the *S. aureus* infection. But taking care of the wound bed and the large amount of exudate showed how passive venting and active microbial sequestration can work together. The Penrose silicone drain is a passive tube that works with gravity and capillary action. The main benefit is that it works better than anything else in the "source control" phase, letting out high-pressure fluid and relieving tension in important tissues [2]. But it does let fluid out without having a built-in way to deal with leftover microorganisms in the area. On the other hand, DACC technology uses physical hydrophobic interactions to trap pathogens. Recent in vitro studies have shown that DACC-coated fibers effectively bind *S. aureus* and inhibit its metabolic activity without inducing bacterial cell lysis [4]. The dressing stops the local release of proinflammatory endotoxins by neutralizing intact bacteria. This is a very helpful mechanism for older people who are prone to too many inflammatory cytokine cascades [4]. The use of this technology on our patient is in line with clinical studies that show DACC cavity dressings work well on very exudative anatomical defects [5,6].

Limitations

Even though the clinical outcome in this case was very good, there are a few things that need to be said about it. The 53% decrease in purulent volume between POD 1 and POD 2 cannot be solely ascribed to the DACC dressing, as this decrease significantly represents the natural post-operative evolution following effective surgical source control and suitable systemic antibiotic treatment. Second, although in vitro models show that DACC can sequester *S. aureus* [4], quantitative post-operative swabs or serial cultures were not routinely collected as the patient demonstrated rapid, unambiguous clinical resolution. This lack of serial quantitative microbiological tracking and continuous systemic inflammatory marker monitoring makes it impossible to accurately measure the exact decrease in local bioburden over time. Consequently, DACC-coated dressings ought to be regarded solely as a supplementary measure for local wound management, rather than an independent clinical intervention for deep fascial infections.

## Conclusions

It is inherently difficult to treat severe DNSIs. There is no substitute for prompt surgical decompression and targeted IV antibiotics; however, clinicians can still improve their management of the local wound environment. In this particular case, replacing a standard passive Penrose drain with an active DACC-coated dressing during the sub-acute recovery phase proved to be a highly feasible and safe adjunctive option, providing a secure and effective means to associate physical bioburden sequestration with the management of substantial, persistent exudate. Of course, one case observation can't change the rules doctors generally follow. In the future, larger comparative trials with strict serial microbiological tracking will be needed to determine exactly how much bioburden these advanced dressings remove in deep fascial planes.
